# How do organisations implement research impact assessment (RIA) principles and good practice? A narrative review and exploratory study of four international research funding and administrative organisations

**DOI:** 10.1186/s12961-019-0515-1

**Published:** 2020-01-20

**Authors:** Adam Kamenetzky, Saba Hinrichs-Krapels

**Affiliations:** 1 National Institute for Health Research Central Commissioning Facility, Twickenham, TW1 3NL United Kingdom; 2 Policy Institute at King’s College London, Strand Campus, London, WC2B 6LE United Kingdom; 3 King’s Global Health Institute, King’s College London, Denmark Hill, London, SE5 9RJ United Kingdom

**Keywords:** Impact, research impact assessment, RIA, research funding organisations, research funders, research evaluation, research assessment, science of science

## Abstract

**Background:**

Public research funding agencies and research organisations are increasingly accountable for the wider impacts of the research they support. While research impact assessment (RIA) frameworks and tools exist, little is known and shared of how these organisations implement RIA activities in practice.

**Methods:**

We conducted a review of academic literature to search for research organisations’ published experiences of RIAs. We followed this with semi-structured interviews from a convenience sample (*n* = 7) of representatives of four research organisations deploying strategies to support and assess research impact.

**Results:**

We found only five studies reporting empirical evidence on how research organisations put RIA principles into practice. From our interviews, we observed a disconnect between published RIA frameworks and tools, and the realities of organisational practices, which tended not to be reported.

We observed varying maturity and readiness with respect to organisations’ structural set ups for conducting RIAs, particularly relating to leadership, skills for evaluation and automating RIA data collection. Key processes for RIA included efforts to engage researcher communities to articulate and plan for impact, using a diversity of methods, frameworks and indicators, and supporting a learning approach. We observed outcomes of RIAs as having supported a dialogue to orient research to impact, underpinned shared learning from analyses of research, and provided evidence of the value of research in different domains and to different audiences.

**Conclusions:**

Putting RIA principles and frameworks into practice is still in early stages for research organisations. We recommend that organisations (1) get set up by considering upfront the resources, time and leadership required to embed impact strategies throughout the organisation and wider research ‘ecosystem’, and develop methodical approaches to assessing impact; (2) work together by engaging researcher communities and wider stakeholders as a core part of impact pathway planning and subsequent assessment; and (3) recognise the benefits that RIA can bring about as a means to improve mutual understanding of the research process between different actors with an interest in research.

## Background

There is an increasing drive for organisations that fund, support and/or administer research (hereafter referred to as ‘research organisations’) to be held accountable not only for various administrative and research governance functions but also for the longer-term impacts of the research that their activities and funding support. This is evidenced by the proliferation of approaches to assess research processes, policies and productivity [1]. The emerging practice of research impact assessment (RIA) is an area where there have been a number of developments – be these analytical tools to help conceptualise impact (in its myriad forms), such as the Payback framework [2], the inclusion of impact as a criteria to determine the allocation of public funds to higher educational institutions (e.g. the United Kingdom’s Research Excellence Framework) or methods to determine the wider ‘spill-over’ effects from government and charitable investments in research, as a means to advocate for the value of the combined contribution of these sectors to national research and development efforts [3].

### What is RIA and why do research organisations have a role to play?

RIA falls within a series of practices referred to – somewhat interchangeably – as ‘the science of science’, ‘research on research’ or ‘meta-research’. The founding Editors-in-Chief of the journal *Health Research Policy and Systems* defined such practices, in their broadest sense, as being “ *… conducted for a variety of purposes, including to strengthen the capacity to undertake scientifically valid and relevant research and to maximise and more equitably spread the benefits that can come from investing in research*” [4].

The International School on Research Impact Assessment (ISRIA) was set up by research organisations who recognised a need for research impact strategies and associated assessment efforts to be given an explicit practitioner focus based on principles of good practice and the application of robust and repeatable evaluation methods. Drawing on insights from over 400 scholars and practitioners attending five ISRIA schools – with stakeholders from research funding agencies, and the health sector as the most highly represented among these – ISRIA developed a series of best practice guidelines [5]. These guidelines aim to distil down ‘what works’ in RIA, and how to situate such practices in a broader organisational context. Essentially, the ISRIA guidelines encourage organisations to (1) analyse the research context, (2) reflect on the purpose of RIA, (3) identify stakeholders’ needs, (4) engage with the research community, (5) choose appropriate conceptual frameworks, (6) choose appropriate evaluation methods and data sources, (7) choose indicators and metrics responsibly, (8) consider ethics and conflicts of interest, (9) communicate results, and (10) share learning.

National RIA exercises tend to define research impact as a change or benefit demonstrably realised beyond academia as a result of research activity(ies) [6]. Most of these RIA exercises, and thus impact definitions derived from them, are driven by research funding organisations or funders of funders (i.e. governmental or other public research funding agencies) [7]. Yet, in spite of calls for RIA to be deployed as a means for robust analysis of aspects such as the effectiveness, efficiency and equity of research [8], most impact definitions exhibit a clear positivity bias, thus encouraging the use of RIA as, at best, a route to bolster organisational advocacy efforts or, at worst, a means “*to count what is easily measured*” [7]. A concern is consequently that research organisations with a role in supporting research and related activities are not deploying RIA with sufficient consideration of its potential to understand the real-world processes, for example, community involvement [9] or engagement activities [10], that might augment research having wider societal impacts. This is borne out by studies of research organisations’ roles. Most organisations do not base their efforts to encourage the translation of research into meaningful impacts on people’s lives around evidence of what works in practice [11]. Studies of funding managers themselves highlight their limited knowledge of complex phenomena such as ‘implementation’, risking a blurring of responsibility and thus hampering any potential facilitative role for research organisations [12].

### There is a need to see how research organisations undertake RIA

To evaluate impact, ISRIA’s guidelines recommend that research organisations use “*a multitude of methods from social science disciplines to examine the research process with a view to maximising its societal and economic impacts*” [5]. Crucially, these guidelines do not advocate for any specific framework but recommend to “*critically choose frameworks in a way that fits the context and purpose of a given RIA exercise and to explicitly state the limitations of the chosen framework*” [5]. Despite the need for accountability of research funds, the growing activities around RIA, and the development of RIA principles and a community of practice (all alluded to above), we do not know if there is empirical evidence that demonstrates if and how such critical and methodical approaches to RIA work in practice within research organisations. Though empirically grounded policy research has informed the setup and operations of integrated public research funding agencies, such as the United Kingdom National Institute for Health Research (NIHR) [13], and national RIA exercises of higher educational institutions, such as the United Kingdom Research Excellence Framework [14], our observations are that institutional/organisational policies for RIA – in particular those of grant-awarding research funding agencies – are lacking in an empirical basis (in common with findings from other studies of research organisations’ practices, as noted above). Our question of interest for this study was therefore to ask what experiences research organisations had in putting into practice RIA activities, frameworks and approaches as well as how these experiences might inform others as they develop policies around impact and its assessment.

Examination of organisational RIA activities in practice, on the ground, is important for a number of reasons. Firstly, much of the scientific literature on research impact is theoretical in nature, with the concept of ‘impact’ itself emergent and complex. Attempts to draw together this literature demonstrate the challenges. In their systematic review for the NIHR’s Health Technology Assessment programme, Raftery et al. describe a wide range of underlying philosophical ‘ideal types’ of impact [1]; they conclude that a logic model approach “*with scope for interpretative case studies to explain unique and/or non-linear influences*” is appropriate for assessing the impact of the bulk of Health Technology Assessment-funded research. They also make the case for further work being needed to determine appropriate models and tools for RIA in other NIHR research programmes.

Secondly, those organisations with sufficient time and energy to engage with the scientific literature will themselves discover many such RIA models and tools but little guidance on what could work and for whom, from the ‘lived’ perspective of those working within the organisation. One systematic review in healthcare research identified 24 unique methodological frameworks for RIA, proposing a total of 80 different metrics [15]. Another narrative review identified 16 different RIA models, also pointing out that a majority of RIAs did not involve policy-makers and end-users of research, and thus risked promulgating a bureaucratic bias to organisations’ consideration of impact [16], compounding issues previously highlighted.

The impetus for our conducting this study has come from initial observations afforded through one author (AK) being appointed as a researcher-in-residence with NIHR to explore questions around impact and routes to embed methodical approaches to RIA. NIHR is the largest public funder of health and care research in the United Kingdom, whose management of upwards of £1 billion annual funding is directed through a series of independently commissioned coordinating centres. Via interactions with staff, including a series of sequential cohorts enrolled into a formal impact training co-designed with colleagues at King’s College London, it was evident that data required to conduct RIAs are hard to come by, exist in a variety of forms, and are not systematically captured or published across multiple NIHR programmes. This is further complicated by a breadth of evaluative approaches employed; within NIHR alone, the range of published results of RIAs include econometric [17–19], case-study based [20], narrative synthesis [21] and documentary review [22]. Additionally, semi-standardised impact data collection systems, such as Researchfish™, have had little empirical validation since being rolled out across multiple NIHR programmes and, indeed, other United Kingdom and international research organisations [1].

Thus, our concern is that research organisations, in spite of having a crucial role to play in setting expectations and procedures around impact, are under-served by much of the ‘science of science’ literature, insomuch as it does not extend to practical application or application within a complex research funding landscape.

Our aim is to address this knowledge and practice gap by describing the experiences of research organisations in putting into practice RIA activities, frameworks and approaches. We examined this by (1) identifying published research that provides empirical evidence of organisational experiences of research impact and its assessment, particularly from the funders’ perspective, and (2) supplementing this with reflections from interviews with a convenience sample of four regional and national public research organisations contributing to international best practice in this emerging field [5].

## Methods

### Narrative literature review

We searched the English language scientific literature in November 2017 with the aim of determining the extent of published empirical observations of research organisations’ experiences of research impact and RIA. Studies of particular interest were those reporting on interviews with or observational/participatory/operational/action (i.e. qualitative) research from the perspective of research organisations undertaking impact assessment. We searched the databases listed below, setting the timespan for the searches to the maximum allowable (noted in years following the name of each database) – AGRIS (18), EMBASE (43), MEDLINE (70), Global Health (47), HMIC (38), PsycINFO (201), and Social Policy and Practice (36). We used a search string modified from Deeming et al. [23], which included terms specific to papers exploring health and medical RIA frameworks. As our intention was to identify literature reporting on the experiences of national/international public (e.g. government, charity, not-for-profit, health and/or general medical) research organisations, we included additional terms to generate a larger initial pool of publications of potential interest (Appendix 1). We excluded studies reporting only conceptual/theoretical impact assessment frameworks, systematic or narrative reviews, and/or studies reporting the results of RIAs that did not include empirical reflections from/observations of the organisations themselves undertaking or commissioning these activities.

Our preliminary synthesis involved reading abstracts and, where relevant, full texts of the studies returned from the database searches and noting whether they met the aims of the narrative review, as described above. We also noted the primary aims and focus of excluded studies.

To aid our description of the extent of literature reporting observations of research organisations undertaking RIA, we grouped findings of included studies under three broad domains of focus relating to the ‘structures’, ‘processes’ and ‘outcomes’ relevant to organisations’ various RIA activities. This approach, set out originally by Donabedian as a means to evaluate healthcare quality and applied widely in health services research [24], is used here as an aid to present key features of studies included from the literature and, subsequently, interviewees’ reflections on RIA within their own organisations. It is not intended as a formal means of evaluating the quality of RIA practices, rather to explore, at a more abstracted level, how RIA is situated within organisations whose roles span various aspects of the health research funding landscape.

### Interviews with research organisations

The second stage involved an enquiry of a convenience sample of representatives from four regional and national public research organisations contributing to international best practice in this emerging field, identified by their participation at the last of five outings of the ISRIA in November 2017. Given the relative newness of RIA as an area of expertise, ISRIA provided a unique opportunity to identify the main research organisations actively engaging in, and contributing to, its practice. We approached four organisations, chosen to represent different global regions and varying levels of experience in conducting RIAs, and note details in Table 1. We conducted four interviews with a total of seven staff (i.e. two joint and two individual interviews), whose roles within their organisation ranged from senior executives for research performance/evaluation/management, research impact management and/or research impact analysis. AK conducted the interviews and was also a participant and part-time facilitator at the ISRIA conference. AK and SHK worked on designing the interview topic guide. SHK was a faculty member at ISRIA.

**Table 1:** Details of ISRIA 2017 faculty member organisations interviewed in convenience sample

Organisation name	Organisation type and/or remit	Year established	Approximate 2016–17 research spend (UK£ millions equivalent)^a^	Website	Area(s) of research spend and/or activity
Commonwealth Scientific and Industrial Research Organisation	Federal government research funder (Australia)	1916	660	https://www.csiro.au/	Cross-disciplinary, supporting activities themed by societal challenge areas
Alberta Innovates	Regional government research funder (Alberta, Canada)	2010 (formerly Alberta Innovates: Health Solutions)	175	https://albertainnovates.ca/	Cross-disciplinary, supporting activities themed by sectoral focus areas
Agency for Health Quality and Assessment of Catalonia	Regional government agency specialising in data assessment and analysis (Catalonia, Spain)	1991	Not applicable (provides analytical and strategic function for regional government research funding)	http://aquas.gencat.cat/ca/inici/index.html	Advisory and strategic role, focussing on health and care research, health technology research, health systems, health innovation, and public procurement
Novo Nordisk Foundation	Independent philanthropic foundation with corporate interests	1927	505	http://novonordiskfonden.dk/da	Underpins commercial and research activities of Novo Group companies, and provides grants to support scientific, social and humanitarian causes

^a^Converted into equivalent UK£ at Sept 2018 exchange rates

We used semi-structured interviews based around a topic guide (Appendix 2). Areas of omission in the literature that limited the practical application of RIA activities (for example, local context, resources and challenges of implementation) formed a particular focus for the questions. We transcribed interviews verbatim and then undertook thematic analysis, grouping themes against the three Donabedian domains of focus previously described [24]. The first ‘structure’ explores themes relating to the setup of the four organisations we interviewed and factors relating to the organisations themselves. The second ‘process’ looks at the assessment activities that organisations carry out to support impact and its assessment. The final domain ‘outcome’ presents interviewees’ reflections on what doing RIA has meant – the value RIA has brought to the organisation, to researchers, and to wider communities and stakeholders.

### Document analysis

Interviews were followed by desktop research for documents relating to each organisation's approaches to impact and its assessment, which included annual/impact reports, published online strategies, and any studies published in the literature (as guided by interviewees, and if not already forming part of the literature search, detailed above).

## Results

### Findings from narrative literature review

Of 129 papers identified using our search criteria, we found only five published examples of research organisations describing and/or reflecting back on their approaches to RIA [25–29]. We have summarised these by Donabedian’s domains of focus in Table 2 [24] and discuss key findings below.

**Table 2:** Published studies meeting inclusion criteria by key domains of focus reported on in the study (per Donabedian [24], as described above)

Short reference	Research organisation forming focus of study	Reflects and/or reports on …		
		structures of RIA?	processes of RIA?	outcomes of RIA?
Searles et al. ‘An approach to measuring and encouraging research translation and research impact’ [25]	Hunter Medical Research Institute, Australia	✔	✔	
Greenhalgh et al. ‘Maximising value from a United Kingdom Biomedical Research Centre: study protocol’ [26]	National Institute for Health Research Oxford Biomedical Research Centre, United Kingdom	✔		
Trochim et al. ‘Evaluation guidelines for the Clinical and Translational Science Awards (CTSAs)’ [27]	National Institutes of Health (CTSA funding programme), US	✔		
Rubio et al. ‘Developing Common Metrics for the Clinical and Translational Science Awards (CTSAs): Lessons Learned’ [28]	National Institutes of Health (CTSA programme), US	✔	✔	✔
McLean et al. ‘Understanding the performance and impact of public knowledge translation funding interventions: protocol for an evaluation of Canadian Institutes of Health Research knowledge translation funding programs’ [29]	Canadian Institutes of Health Research (knowledge translation funding programme), Canada	✔	✔	

#### Structural aspects of RIA reported in the literature

Searles et al. present a conceptual model to support and evaluate impact at Australia’s Hunter Medical Research Institute [25], which considers the likely resource intensity of different evaluation frameworks, having systematically compared their various capabilities [23]. They explicitly set out a dual purpose both to support processes of ‘research translation’ (which they provide a working definition of) and measuring ‘research impact’ (also defined and tailored for health and medical research). Their prototype ‘framework to assess the impact from translational health research’ focuses on a (micro) research-level modified programme logic model – a blend of ‘Payback framework’ domains [2], social return on investment and case studies. The authors recognise that their model is as yet untested, though they reflect thoughtfully on potential opportunities and issues relating to its successful implementation (discussed further in ‘processes’, below).

Greenhalgh et al. set out the United Kingdom NIHR Oxford Biomedical Research Centre’s plans to apply an evidence base to – and research how – regional partnerships between universities and healthcare organisations play out with respect to the Centre’s ambitions of translational research [26]. As part of a protocol to ‘maximise value’, they propose future RIA activities designed around organisational case studies, informed by action research. As with Searles et al. [25], above, the protocol is untested; nonetheless, the authors set out a number of wider contextual aspects relating to the wider research funding environment, governance, collaboration and resourcing that underpin the proposed initiative and the work of the Centre more broadly. They also note a series of operational objectives that will form the basis of future RIA activities, including establishment of a ‘partnerships’ external advisory group and associated stakeholder engagement activities, and use of research on research methodologies to evaluate progress and impact.

Trochim et al. present a series of principles to guide evaluations of translational biomedical research [27], building on their previous work such as the Evaluation of Large Initiatives project, which had been designed to evaluate research of a large centre funded via the US National Cancer Institute [30]. They reflect on the importance of high-quality research evaluations, and set out key issues and practices to guide the community ‘during evaluation planning, implementation, and utilization’ for the National Institutes of Health (NIH)‘s Clinical and Translational Science Awards (CTSAs). They highlight a number of factors relating to the organisational structure of the CTSAs, including linking evaluation to formal planning cycles, local pilots of smaller scale but nonetheless rigorous approaches, and considering how to integrate RIA at differing organisational levels (e.g. local and national) across NIH’s CTSA programme. While they consider a number of nuanced aspects of evaluation pertinent to RIA more broadly, these are set out as future-looking recommendations – the authors report ‘lived’ reflections on the processes and outcomes of RIA in the CTSA programme in a separate, later paper by Rubio et al. [28], summarised below.

Lastly, McLean et al. [29] reflect on specific objectives (e.g. learning and development, accountability, resource constraints) that are addressed by their protocol to evaluate the Canadian Institutes of Health Research (CIHR)’s knowledge translation funding programmes. They describe a novel method of “*participatory, utilization-focussed evaluation*”, a methodology aligned with the principles of “*integrated knowledge transfer*”, which formed the topic of the evaluation itself. In particular, they focus on the efficacy of participatory evaluation, as judged by those who will use its results (described further in ‘processes’, below).

#### Processes of RIA reported in the literature

Searles et al. [25] describe ‘how’ questions around impact and its assessment as partially informed by a steering group, established to look at issues of bias, communication of findings and scaling issues. The steering group provided a platform for co-design of the prototype framework and was made up of a blend of researchers, clinicians/healthcare staff, and funder and university administrators. The authors note future stakeholder engagement activities as an important feature of the framework’s aim to encourage translation – by defining impact aims and determining relevant metrics for RIA, including process metrics, as part of a dialogue with researchers. To encourage such a dialogue, they propose combining RIA results onto a project scorecard that acts as a record of impact as the research progresses. The authors provide hypothetical scorecard examples, given that the framework was yet to be put into practice.

In the literature relating to assessment of the NIH CTSAs, Trochim et al. [27] had previously set out a number of nuanced aspects relevant to consideration of evaluation methodologies, uses and policies, including guidance on how to build capacity for the development of RIA as a practice within NIH. Of particular note were aspects relating to the scope of evaluations, including stakeholder engagement, scale, professional standards, and intensity of resource and ambition required to evaluate innovative programmes. Reflecting back on these activities following a pilot exercise to develop a common set of metrics across the CTSAs, Rubio et al. [28] – notably, the only group we found to have published reflections on lessons learned subsequent to undertaking RIA activities (noted further in ‘outcomes’, below) – describe their strategy for engaging with individual CTSA institutions via their participation in a Common Metrics Workgroup. This Workgroup was a subgroup of a CTSA-wide Evaluation Key Function Committee, made up of evaluators from all 62 CTSAs. Key factors noted as important to the effort to develop common metrics were to prioritise those that were of low burden to both researchers and the CTSA, but high value to the research institution and the CTSA. They also recommended working iteratively, using formative evaluation methodology, to pilot and revise individual measures with regular feedback (e.g. surveys and conference calls) from those collecting data.

McLean et al. [29] reflect on the need to collaborate with multiple stakeholders, with a view to improve the ultimate use of RIAs by end-users. They propose multiple methodologies to elucidate qualitative and quantitative evidence on CIHR’s role (in this case, enabling and promoting knowledge translation) as well as activities to situate CIHR with similar organisations around the world. They also propose an expert review panel to offer an independent opinion on activities and analysis of the Evaluation Working Group. Subsequent web searches revealed this group had published an evaluation of CIHR’s Knowledge Translation Funding Program on CIHR’s website [31] which, while not (strictly) meeting our inclusion criteria for publication in the scientific literature, we felt worth including in our sample of organisations taking an empirically robust and reflexive approach to RIA.

#### Outcomes of RIA reported in the literature

Rubio et al. [28] were the only group to report observations across all three domains of focus – structure, process and outcomes – relating to their experiences of establishing and piloting a common approach to metrics for RIA, across the portfolio of NIH CTSAs. They note success in that the pilot identified a number of metrics that could be consistently reported despite CTSA institutions having different legacy processes and data systems. This provided a template for further efforts to simplify and reduce the burden of RIA. They also note the value of having taken a systematic and methodical approach to developing common metrics as providing an ‘empirical anchor’ to use as the basis for more in-depth evaluations of CTSA performance and their role in underpinning research translation. The detailed and reflexive nature of this and the previous Trochim et al. study [27] noting intended evaluation principles and approaches for the CTSAs would seem particularly relevant to governmental/federal funders of (especially biomedical and health) research with an interest in evaluation, and we explore a number of aspects raised by this group further in our discussion.

The remaining 124 studies identified in our search did not meet our inclusion criteria given that they reported only the results or outcomes of RIAs rather than the organisational processes of undertaking and/or learning from RIAs; presented generalised reflections on features of RIA frameworks, approaches or activities from an external perspective (i.e. academic or consultancy role), rather than any ‘lived’ practical application; or reviewed RIA approaches with the intention of applying them in an organisational setting, but presented no empirical data or perspectives from the organisation(s) themselves.

### Findings from research organisation interviews

The findings from the narrative review confirm that there is a relative paucity of empirical data in the published scientific literature that looks holistically at features of the research organisations themselves doing RIA as well as the theory, design and results (i.e. outputs) of RIA studies. Thus, we present results from the second stage of the research, namely findings from the interviews with a convenience sample of research organisations contributing to international best practice by virtue of their status as faculty members of ISRIA.

#### Structural factors relevant to research organisations’ RIA practices

Common across all interviewees was the notion that RIA practices were in their infancy, and we observed varying levels of maturity with respect to structural set ups for conducting RIAs. Interviewees described a number of systemic ‘rate limiting’ factors contributing to the success of efforts to implement and scale up robust RIA processes. In particular, these include support from senior management and strong leadership, developing a skills base for evaluation, and automating data collection wherever feasible.

##### Senior management support, leadership and resourcing of RIA

A key factor relating to the organisational structures that supported RIA was the aspect of leadership.

Research organisation #2 reflected on the importance of having a well-respected leader acting as a spokesperson for more rigorous and comprehensive approaches to RIA. They felt that “*the right people and right drivers at the right time*” helped them to make headway. But leadership for impact was more than just a ‘top down’ exercise, as highlighted by this quote:

“*Managing upwards is exhausting. Trying to keep impact on the radar of the executives, of the board, of the CEOs, and them understanding what the hell impact is, how it links back to the core person* [within the organisation] *… that's exhausting and has massive challenges. Because they're distracted by everyone else throwing their framework, their idea, their area of research, or their area, even, of other support services trying to stay on the radar.*” (Interviewee A)

Research organisation #2 noted that setting up RIA activities required senior management to create conditions that would allow for constructive engagement with research communities (we discuss engagement under ‘processes relevant to RIA’, below). When it came to scaling RIA activities up, strong leadership (noted by research organisations #2 and #3) and appropriate resourcing (noted by research organisations #2, #3 and #4) appeared to be common determinants of research organisations’ abilities to meet demands for the RIA data.

##### Developing a skills base for evaluation

Business models for conducting RIA varied both between and within research organisations, with a number of different approaches in place. These ranged from external commissioning of evaluations through to internal programme reviews. Which evaluation model organisations used was determined by having the right skillset for conducting RIA, which varied considerably from organisation to organisation. We know from another study looking at the capabilities of research organisations using RIA data that, while larger organisations may have in-house evaluation and analysis teams to produce analytical reports, it has taken them considerable time to develop this capacity and capability, and such resources may not be available to smaller organisations [32].

Independent validation was an important factor to those we interviewed in providing rigour, though it could be costly. Research organisation #4 noted that they worked with external consultants in a collaborative fashion, such that in time they could ultimately bring elements of these analyses in-house.

A strategy to reduce consultancy costs was illustrated by interviewee A’s description of commissioning economic impact assessments via procurement-approved panels of impact consultants. They described a model whereby they initially paid external evaluators upwards of £50,000 to generate detailed, mixed-method case studies (i.e. combining qualitative evidence and econometric data) of the impacts of specific research investments. Having taken the time to standardise methods for generating these case studies, and train staff accordingly, the organisation reduced the cost of each case study to under £15,000. Internal teams now work with researchers across a ‘pipeline’ of impact-related activities (be these planning, monitoring or evaluation), while gathering novel data to feed into downstream case studies, applying the same standardised methods.

##### Automating data collection wherever feasible

A major structural factor that facilitated cross-organisational RIA activities was the availability of records – and in particular well-curated electronic records – to identify research topic areas, extract data and aggregate these for the purposes of assessing impact.

Organisation #2 identified the administrative challenge of manually retrieving studies against a particular topic. They recognised the value of algorithmic and semi-automated approaches such as Digital Science’s Dimensions tool, to help search for and validate records in a particular research domain or theme, before going on to query the extent and availability of impact data to analyse manually.

Two research organisations (#2 and #4) used Researchfish® (an online platform that enables research organisations to capture and track the impact of research investments and/or activities, and enables researchers to log the outcomes of their work) as a means to capture and categorise research outputs electronically. However, research organisation #2 made a point that their use of this electronic tool was only a relative success as a result of efforts to engage their local research community around the purposes and principles of undertaking RIA across their funding portfolios. They pointed out that researchers may not always report on their impact activities adequately and so engagement with them was necessary (more on this in ‘engaging researcher communities’, below). They noted that acceptance of reporting on impact and contributing to the organisation’s RIA, was in part due to a rollout of Researchfish®, which included acting on feedback from researchers by, for example, acknowledging that researchers were concerned of administrative burden of reporting, and making this process easier by not requiring them to continue reporting through old written annual reports once the system had been implemented.

#### Processes relevant to RIA

Under processes we include research organisations’ accounts of the activities that they carry out for RIA – from grassroots efforts to engage researcher communities to articulate and plan for impact, using a diversity of methods, frameworks and indicators for impact assessment, and supporting a learning approach.

##### Engaging researcher communities to articulate and plan for impact

All research organisations spoke of RIA as a highly collaborative practice that ought not to be undertaken without thoughtful and sustained efforts to engage with the researcher community and others – both internal and external to their own organisations – with an interest in the research being assessed.

Reinforcing this were interviewees’ descriptions of embedded activities, designed as an integral part of research planning and activity monitoring (i.e. not just for the purposes of assessing impact). Throughout, it was apparent that these activities required dedicated time and resource, not least staff with sufficient skills, to run them – and we reflect further on resource implications for research organisations below.

At the earliest stages of the research process, research organisation #1 spoke of routinely conducting workshops involving the research team, ‘end-users’ (i.e. those whom the research is intended to involve and/or benefit) and others stakeholders, to help them articulate their intended impacts. Research teams’ willingness to involve a suitably diverse stakeholder group in these discussions was used as a heuristic for whether they were ‘RIA ready’ (i.e. whether it was yet appropriate for them to consider being part of more formal evaluations, requiring more than process and activity data collected by the organisation as part of their standard portfolio monitoring).

As another example, they described an instance where researchers working in the manufacturing sector were initially reluctant to describe the potential for health benefits from their work, not themselves having the expertise to evaluate impacts in this field. They spoke about how they supported these researchers to articulate these impacts, and offered resources to help future evaluation in these domains:

“*It was trying to push them, going, ‘Actually, there are indicators that we could use. You don't have to be the one that collects it. We can either get a social scientist, or sometimes, the organisation itself collects that information.’ So it was trying to wean them off thinking that they were the only ones that had the right to collect the information, and to go into areas that weren't their area of expertise as well*.” (Interviewee A)

A telling lesson – particularly relevant for research organisations looking to embark on formal RIA exercises for the first time – came from this same interviewee reflecting on the need to bring researchers along with any strategy, rather than impose it on them:

“*If we're going to do a post-assessment of any of our projects, at the moment, they were never set up and designed to be actually monitored in reporting impact. So you'll find it can be a little bit hit and miss on what type of data they collected along the way, and what type of evidence to the claims that they're making, and all those types of things*.” (Interviewee A)

Research organisation #3 noted how transparency in their approach to RIA was a motivating factor for many researchers, who had responded enthusiastically to the opportunity to have their work form part of a snapshot of research activities. They noted that this was particularly the case for early career researchers, whose work tended to be less well represented by ‘traditional’ (mostly citation-based) assessment metrics.

##### Using a diversity of methods, frameworks and indicators

Despite aspirations to develop a ‘common language’ with which to explain organisational impacts and approaches, comparing across the research organisations we interviewed, it was striking how different these approaches appeared. RIA methods (or perhaps more accurately, methodologies) ranged from regular survey-based assessments of whole portfolios of research, to populating logic models with routinely captured programme outputs, to externally commissioned evaluations in specific domains of impact (e.g. cost-benefit analysis), to in-depth case studies co-produced and guided by research teams according to the availability of RIA data in their field.

Interviewee responses all indicated that managing a hierarchy of RIA activities, i.e. determining the ‘unit of analysis’, and determining which research projects/programmes are responsible to report into said analysis, was not a trivial task. They reported pragmatism, as much as any formal methodology, in guiding their initial efforts.

There was recognition by all interviewees that a diversity of approaches was needed for RIA, highlighted by the following quote:

“*No one system is going to answer all of the questions and meet all your needs*." (Interviewee C)

Research organisations variously noted the value of RIA frameworks and indicators as a means to help situate conversations with research communities, communicate with other funders (if funding in similar areas of research) and benchmark between organisations’ activities. While all interviewees drew on published literature as the basis of their approaches – from formal bespoke RIA frameworks, to broader conceptualising tools such as the use of programme logic models – two research organisations described additional activities that our analysis finds less well reported in the literature, that we note below.

Research organisation #1 spoke of ‘learning by doing’, taking an established RIA framework, approach or method and adapting it through experimentation and discussion with the research community.

Research organisation #2 described initial data audits (as opposed to more formal ‘research on research’ studies) as a means to use existing reports, grant data and other statistics to populate a draft RIA framework; this could be at the project-by-project or programme-by-programme level. Starting off with a limited – if imperfect – number of impact categories or indicators, nonetheless derived in an iterative and reflexive fashion, could overcome ‘death by indicators’, as described by the following interviewee:

“*Where do the indicators end? It can be overwhelming, if you say: ‘You might have some social impacts. Do you want to open up the Excel spreadsheet that has a look at social indicators?’ – and there are 5,000 of them. They're going to shut it and not even engage with it*.” (Interviewee A)

Research organisation #1 also described the value of a framework in terms of expressing the organisation’s main areas of focus for RIA (e.g. economic, social and environmental impacts), with indicators helping to progress a more granular conversation with researchers around the kinds of activities they might be in a position to report on (as opposed to those that would require more in-depth and bespoke evaluative activities to capture).

They also described the value of workshops to help populate an organisation-wide impact framework or test the appropriateness and usability of existing frameworks and impact indicators across multiple funders. These activities, conducted transparently and with the intention of engaging wider communities at their core, ensured that impact indicator sets (as well as organisational ambitions) had a degree of legitimacy with different stakeholder groups – helping ambitions, as noted above, to develop a ‘common language’.

##### Supporting a learning approach

Looking outward, international engagement with other funders helped organisations to learn from each other, as part of a ‘community of practice’, ultimately connecting different actors across the global innovation system.

Two research organisations (#1 and #2) noted the value of asking other funders ‘*How do you do it?*’, as a means to move RIA into a more mature level. Research organisation #2 described efforts to set up a series of workshops, based around the ISRIA core teaching materials,^1^ to bring together different actors in the funding community and across other sectors, as part of a peer-to-peer learning process. They noted a major driver being an aspiration to develop common RIA languages and tools:

“*A lot of the organisations are being asked the same questions. It only makes sense to put our minds and our experiences together to figure that out, because there is no clear path forward, necessarily*.” (Interviewee C)

Having a set of publicly available RIA guidelines provided a means to ensure research organisation #1 applied a consistent approach, while embedding learning in evaluation techniques and approaches more widely:

“*If you are to conduct an impact case study within* [the organisation]*, it will follow these guidelines, otherwise we won’t recognise it as an impact case study. We've just come together to go, ‘Now, what have we learnt? How can we update the guidelines where we thought this was a particular method we should be using? What other information can we be adding?’ and those types of things*.” (Interviewee A)

Whether inward or outward-facing, all research organisations recognised the value of a learning approach to inform an aggregate picture of research impact, given that neither they nor others in the wider research community operated in isolation.

#### Outcomes of conducting RIA activities

In this last section we report on reflections on what doing RIA has meant to research organisations – the value RIA has brought to the organisation, to researchers, and to wider communities and stakeholders. Interviewees noted RIA data as supporting a dialogue to orient research to impact, underpinning shared learning from analyses of research, and providing evidence of the value of research in different domains and to different audiences.

##### Supporting a dialogue to orient research to impact

Research organisation #3 spoke of the transformative nature of RIA efforts and narratives as “*orienting research to impact, advocating for a different way to design research*” (Interviewee D).

Research organisation #1 spoke of a systemic shift in impact from the being the focus of centralised assessment exercises, to being part of a more informed dialogue between researcher and funder throughout the research lifecycle:

“*Now* [the research teams] *are really starting to link impact clearly to their strategy. They've got a person that works with all their teams to do their impact pathway planning. Not just from my* [central] *team; they've assigned a full-time person to do that. They then work with my team to be checking that they're doing the right thing. So you've got them investing time, effort, strategic alignment that goes beyond my team having to do it for everyone*.” (Interviewee A)

While research organisation #2 felt the proposition of RIA as a means to optimise funders’ return on investments was still somewhat aspirational, they reflected that having impact as a high-level ‘performance indicator’ could help research teams to focus on the capabilities they needed to have an impact, rather than the limitations of their current capabilities. In terms of business development, this helped them to have more realistic conversations around resources required to deliver grantees’ research ambitions – though they also noted that this seemed to be a sensitive subject for a majority of researchers.

Regardless of the mode of assessment, a strong emerging theme was the value of research teams themselves taking ownership of impact plans and ambitions as well as assessment. By linking the organisations’ evaluative practices to researchers’ own strategies, a majority of research organisations (#1, #2 and #3) provided upfront support for impact pathway planning as well as downstream support to identify the availability of impact data and appropriate methods to source it. For these organisations, engaging with research teams to help them see the value of RIA indicated a ‘culture shift’ away from centralised performance management to a more self-motivated and participatory mode of evaluation.

##### Underpinning shared learning from analyses of research

All research organisations stressed the value of regularly collecting and making publicly available RIA data, to share what they were learning from their analyses of research activities.

Basic questions of accountability – answering questions such as ‘*How much did we invest in X, and what did we do?*’ – determined organisations’ early decisions on the appropriate systems to collect and link impact data.

Research organisation #2 described an initial process of review and categorising ‘known’ outputs from research across various programmes, as a first step, before undertaking analysis of impact at a more systematic level. One described a series of programme reviews, designed to feed data into a common impact ‘architecture’ – in their case, a series of programme profiles to commonly describe the duration, objectives, levels of investment and intended outputs for each research programme.

Research organisation #4 described a twofold pathway by which their organisation was introducing RIA practices; firstly, a pragmatic exercise to capture and publish data from annual accountability surveys that could feed ‘rather quick and dirty’ analyses for decision-makers and, secondly, a prospective strategy for more analytical assessments.

An example of more in-depth analytical work by this organisation was their exploration of the nature of researchers’ collaborations with industry. Informed by descriptive statistics initially collected via annual reporting cycles, the research organisation had designed a more detailed analysis (involving semi-structured interviews and combining their datasets with their national register of companies), through which they might learn about motivations of researchers to work with industry (and visa-versa).

Reflecting on value to the research system itself, all interviewees noted that RIA data could help researchers learn how to have a greater impact. Down the line, research organisation #2 noted that sharing the results of RIAs engendered a greater spirit of cooperation from researchers, as part of a virtuous circle:

“*I think it is really important to ensure that we are sharing back with our stakeholders and the research community* [ … ] *We are in this together. If we collect data and we never report back, it is really not much of a collaborative relationship. It’s important for us to share the results that we are achieving, not just for us, but we always acknowledge the efforts that the researchers make. This is their work that we simply help fund and support*.” (Interviewee C)

##### Providing evidence of the value of research

All interviewees indicated that RIA data was providing underpinning evidence to communicate the value of research, in a number of ways.

In the case of research organisation #1, their efforts to standardise methods to generate return on investment data meant that they were now able to calculate an aggregate figure representative of the organisation’s overall return on investment, across its entire research portfolio. This figure, calculated every 2 years, was now being released publicly by the Chairman of the Board and CEO. They noted the value of RIA data in supporting wider organisational engagement activities, particularly involving their organisations’ communications teams. They described how impact-led communications were themselves more engaging – one noting that their organisation could not “*pump them out fast enough*” for the demands of their communications team and ministerial liaison office.

‘Soft’ advocacy formed the principal reason to scale up RIA practices in this organisation. They couched this not in terms of direct appeals for funding, but instead being able to provide a more robust series of answers to generic questions such as return on investment or the contribution of research investments in different thematic portfolios (e.g. health, environmental sciences, engineering) to specific issues forming the focus of public policy initiatives (e.g. tackling CO_2_ emissions). They noted RIA as a means to generate “*the feedback that’s required in regards to decision-making*” as well as “*accountability back to the public on what we’ve spent their money on, and what they’re getting for it*” (Interviewee A).

Interestingly, none of the research organisations we interviewed indicated that their RIA activities nor the evidence they generated were yet sophisticated or systematic enough to justify high-level decisions around the allocation of research funds. Indeed, research organisation #2 stated caution in responding without due care to what they perceived as an increase in demand for questions around how best to allocate public resources for research:

“*If around the world there is not strong evidence in research and innovation, or science, supporting how to allocate investments using any credible good designs or evidence, then we have to identify the imperfect science for that aim. Then, as a community, if the decision-makers and policymakers are asking more for it, we have to figure out, as a community, how we are going to respond to that demand. We are treading extremely carefully in terms of allocation. Extremely*.” (Interviewee B)

Thus, while RIA was recognised as providing one line of evidence to help inform programme decisions, it was by no means the only evidence that could do so – stakeholder consultation was recognised by a majority of research organisations (#1, #2 and #3) as crucial to exploring the consequences of RIA data being used in future allocation decisions, such as that undertaken by the United Kingdom’s Research Excellence Framework.

A spirit of cooperation or shared endeavour in RIA had the potential for research organisations and researcher alike to make the case for continued investment in research – and thus act in concert to benefit society, as expressed by the following interviewee:

“*I think, we are very fortunate that our research community, by and large, understand the need to start demonstrating impact, and that we are in this together. That we fund them. They generate outcomes and impact. That we need to show what our researchers are getting, so that we can advocate for continued funding from the government. That it is not them in isolation, or us in isolation. That we really do need to bring our efforts together*.” (Interviewee C)

## Discussion

Our aim in conducting this study was to describe the experiences of research organisations in putting into practice RIA activities, frameworks and approaches. We found that the scientific literature on impact, though containing a number of examples of RIA practices of potential relevance to research organisations, was abstracted from the realities of organisations’ lived experiences. By comparing observed sets of RIA structures, processes and outcomes from our interviews with what is represented by the limited (*n* = 5) examples of experiences published in the scientific literature, we can begin to suggest what might be considered good ‘realistic’ practice in this highly emergent field. We thus set out three high-level recommendations for research organisations derived from our analysis and linking to the Donabedian domains of focus [24] – *getting set up* for RIA, working with *diverse stakeholder groups* to plan for impact and its evaluation, and *realising the benefits* of RIA as a means to underpin shared sectoral learning.

### Get set up: impact strategies need leadership, skilled evaluators, effective data systems, and time to set up and deliver

Our overarching impression from the accounts of all interviewees was that organisational maturity and readiness for RIA varied considerably within each organisation, not least across the four different organisations in our convenience sample – all of whom were already part of engaged learning activities through their participation at ISRIA. Interviewees described various structural aspects that could be considered ‘rate-limiting’ factors to developing impact strategies and, within this, the design and delivery of RIA. Thus, our first recommendation calls on research organisations to consider carefully and prospectively the structures within their own organisations that may facilitate their future capacity and capability to undertake RIA.

Two research organisations we interviewed noted gains to be made from semi-automated methods to search, validate and analyse impact data. However, they noted a limiting factor to be the extent and availability of electronic records. How to make best use of existing systems and consider efficiencies in data analysis to service a range of potential evaluation questions, would seem a critical point of reflection. In their guidelines, ISRIA make the point that RIA practitioners themselves need to have a nuanced understanding of the merits of different approaches to evaluation, to gather data via methods that address assessment questions efficiently and effectively [5]. Trochim et al. reinforce this point in their broad-ranging series of guidelines for the NIH’s CTSA initiative, reflecting that evaluators ought to aspire to high professional standards of practice and be sufficiently skilled to apply innovative approaches [27]. However, they make the point that, to bring in and/or train evaluation professionals to this level, requires upfront allocation of resources. We observed that research organisations’ own resource constraints for conducting evaluative activities was a critical factor in their scale-up of RIA.

Looking to RIA skills, our interviewees reflected on a number of business models applied to undertake RIA, and the advantages of encouraging a more decentralised (i.e. collaborative) approach. Yet, only one interviewee noted efforts to train staff within their organisation. As part of a team responsible for co-developing an impact training programme for staff working across the United Kingdom’s NIHR, we have observed first-hand the importance of efforts to develop skills and competencies when it comes to exploring questions around research impact and its assessment. ‘Impact literacy’ as a concept has indeed been noted by others working to advance institutional practices in this area, particularly to ensure that approaches (either inherently, or as applied at an organisational level) do not encourage instrumentalism or short-termism [33].

While we would encourage further necessary reflection from research organisations to understand their capabilities across the above important structural domains of RIA, we consider an overarching – and potentially underrepresented – issue to be that of leadership. This was noted by a number of our interviewees as vital for making headway in organisations adopting more rigorous approaches to RIA. Trochim et al. are clear in their recommendations that evaluation practices are integrated into research programme planning and implementation [27]. While they note that responsibility for timely and high-quality evaluation lies across all stakeholders, they particularly highlight the role of research programme leaders – both at the level of the funding organisation and at local research centres – to embed evaluation as “*an ongoing function distributed across all cores*”*.* In the case of the CTSA programme, such an evaluation function was prospectively and explicitly mandated by NIH at the commissioning stage, to be planned and costed into requests for funding by prospective centres, as part of the application process [27].

Taking just these three structural factors – better impact data, RIA skills and leadership – we note the importance of considering the resource requirements that an embedded, strategically relevant and methodical approach to evaluation demands if good RIA practices are to become integral to effective research programming. While we agree with the warnings from a number of commentators against the dangers of research organisations taking an overly bureaucratic approach to impact and its assessment [7], we find as concerning mounting evidence that a number of major RIA programmes are insufficiently resourced to achieve their intended objectives [34] or impact being overlooked entirely as a focus of evaluation by research commissioning programmes operating in the translational/implementation space [35]. While we recognise our own institutional biases (working as mixed methodologists in policy analysis – itself a fairly ‘blended’ research area) we would stress the effort and commitment required to introduce what can be seen as unorthodox methodological approaches to large organisations with well-established working practices. The work of Swan et al. sets out a cautionary tale – they describe how dominant institutional, so-called ‘mode 1’, logics can prevail over and ultimately lead to the abandonment of initiatives that deliberately try and set a focus towards use-led, so-called ‘mode 2’, research [36].

We reflect that structural factors played a large, if not dominant, role in organisations’ early efforts to undertake RIA. These lead us to recommend that research organisations consider their own maturity, and ultimately capability, when determining the purpose and scope of RIA. Ensuring complementarity between RIA activities at different levels of a complex programme(s) of research requires effort to plan and coordinate, if RIA is ultimately to meet the needs of stakeholders with different interests and expectations, as we describe further below.

### Work together: engaging researcher communities and wider stakeholders should be at the core of impact pathway planning and RIA

Engaging with research communities and wider stakeholders, including other funding agencies and government departments, is important for a number of reasons, in particular to help the quality of RIA impact data reported by researchers, for research organisations to better understand what impact means in their research ecosystem and, not least, to address the ethical considerations of collecting impact data.

Interviewees described a range of thoughtful and sustained efforts to engage within and across their organisations’ teams, with other research organisations, and a range of stakeholders with an interest in the research being assessed. These activities ranged from means to encourage peer-to-peer learning of appropriate RIA methods and approaches between organisations, to platforms to motivate research groups to plan and articulate their ambitions for impact. Interviewees also described a spectrum of enabling work for RIA that spanned from inwardly focussed efforts to gain managerial support, to outwardly focussed efforts to promote a cooperative ethos among research teams being assessed, such that they guided the organisation as to which data they were in a position to collect as part of an ongoing dialogue around anticipated or actual impacts that their work had contributed to.

ISRIA’s guidelines set out a number of practical steps that encourage processes of identifying and engaging with stakeholders, and reflecting on their interests, as critical steps in RIA [5]. The authors note that doing so can support the social robustness of knowledge derived from RIA and, by extension, the science that it represents. They also point out that given limited resources for evaluative activities, stakeholder engagement can help to prioritise areas for RIA. Yet, warning signs are apparent from other commentators of the dangers of instrumental approaches to engagement. Jude Fransman, in her nuanced and comprehensive synthesis of the history and ecology of research engagement practices, points out the dominance of academic conceptualisations of engagement [10].

Indeed, we observed that the process of conducting RIA activities in itself supports a dialogue between researcher and research organisation to orient research to impact, based on co-ownership of impact plans and a focus on shared capabilities to deliver and evaluate impact. McLean et al., in their study of research organisations’ roles in translating research, speak of the power held by funders, in particular, in stimulating and incentivising action among the wider research community [11]. This power ought to be used with careful reflection by funders on their motivation for conducting RIA. Mclean et al. make the case that under-investment in critical reflection is not a sustainable means for research organisations to cut costs [11].

In their guidelines for effective RIA, ISRIA highlight the need for clarity from research organisations on their rationale for assessment. Researchers’ perceptions of RIA matter; research organisations ought to consider the ethical implications of their requests for information, particularly where assessment might create perverse incentives (e.g. if linked to further funding or other conflicts of interest). Funders in particular must recognise and mitigate any ‘conflict of commitment’ arising from the time and effort spent by researchers in responding to requests for information [5].

This sentiment is echoed and expanded upon by Trochim et al. [27], who recognise that setting out the implications of RIA can support policy and action as well as clarify conceptual concerns and engage thinking amongst researchers. They encourage research organisations to work collaboratively with local groups and explore the merits of different approaches to evaluation. In their view, the role of research organisations in this area is to provide general guidance, not explicit requirements, to allow scope for local ownership and contextually relevant planning of evaluation activities to take place. Nonetheless, they encourage research organisations developing written evaluation policies or guidance to ensure that these address important topics over and above management and methods, such as goals, roles, participation, as well as the use, dissemination and meta-evaluation of such policies. They reinforce that metrics alone do not make for good evaluations and recommend piloting small, rigorous sets of definitions, metrics and measures. We would echo calls for research organisations to be aware of and adopt, where practical, calls for responsible metrics [37].

Finally, we found that a learning approach, through international collaboration and the sharing of emerging RIA practices, helped research organisations to apply more mature methods and generate better (i.e. more rigorous and more strategically relevant) impact data. This is perhaps not surprising, given all four interviewees were faculty members of a collaborative international programme dedicated to “*learning to assess research with the aim to optimise returns*”. Indeed, a call for mutual learning with the RIA community forms one of the ten guideline points published by this group [5]. A logical first step ought to be mapping the context for evaluative activities – a ‘macro’ example comparing United Kingdom versus Australian perspectives on RIA being that of Williams and Grant [38]. This can help understand the wider environment for RIA and benchmark strengths and weaknesses of the research environment.

### Recognise benefits: a focus on impact can lead to greater engagement between research organisations and researchers, improved communications and ultimately better evidence on the value of research

Throughout our interviewees’ accounts, we noted themes of organisational improvement and benefits to the research system brought about by taking an open, reflexive and methodical approach to RIA. We feel it is necessary to present some of these benefits, and encourage other organisations to do likewise, in the spirit of ‘continuous improvement’ that seeks to improve research funding practices for wider societal gains [39].

Proximal benefits of RIA included research organisations’ access to data that underpinned a learning and cooperative approach to achieving wider impacts. Interviewees spoke of ‘bringing the community along’, echoing calls to improve the social robustness of research and the social desirability of impacts, by involving public and other stakeholder groups with an interest in research throughout the process of impact delivery and impact evaluation [5, 40]. Evaluation processes and their results ought to be open and accessible [27]. Thus, RIA, by setting out methodical and transparent approach to research evaluation, ought to help serve organisations seeking a diversified communications offering, where no one form of impact is preferred over another [5].

More distal benefits of RIA included better evidence of the value of research to inform decisions and ultimately organisations’ ambitions to optimise returns. All of our interviewees reflected on the emergent and relatively new practice of RIA but spoke of this as a learning opportunity. Our own experience in designing training for the United Kingdom NIHR is that research staff are responsive to training on impact and RIA methods, part of a wider programme of work to build capacity in this emerging area. In their guidelines to the wider community, ISRIA notes that a responsible approach to RIA provides one line of evidence by which organisations can make better decisions [5]. Experimentation and variation of approaches to RIA is appropriate: research organisations need not be put off by encouraging smaller, localized efforts that can act as incubators or testing grounds for larger/macro approaches [27]. ‘Joining forces’ via fora to bring evaluators together is one way organisations might tap into the heterogeneity of approaches in this emergent field, noted by Kane et al. as both “*a liability and a strength*” [41]. Softer approaches for knowledge exchange between organisations, such as a series of ‘impact coffee clubs’ set up by the United Kingdom’s Association of Medical Research Charities and the NIHR, could also act to share learning and grow organisational capacity in RIA [42].

Thus, our findings echo calls by others tasked with exploring and mapping emerging practice in this area: making the case for the need for investment to support methodological innovation in research evaluation, the better use of existing datasets, and the wider education and cultural change of the research sector as a whole, if we are to understand the benefits that arise from research and, ultimately, set in place policies to realise the full value of public investments in R&D [43].

## Limitations

In conducting this study, we recognize the emergent and relatively under-explored aspect of research organisations’ own impact practices. We did not search grey literature sources systematically, and appreciate that this body of work may well include results from (and potentially reflections on) methodical and robust RIA activities. However, our aim was to identify the extent to which empirical data and reflections on research organisations’ own impact practices were being reported in the scientific literature, and supplement this with our own access to organisations willing to go ‘on the record’ with their own lived experiences.

Our use of interviews was designed to provide illustrative case studies from a convenience sample of research organisations contributing to emerging RIA practices, and thus not necessarily representative nor appropriate to generalise to research organisations in other contexts (for further discussion on merits of case study method and its value in generating practical knowledge, see Flyvbjerg [44] and, as specifically applied to a study of research impact, Greenhalgh [45]). We sought to apply rigour in how we conducted and analysed interviews, ensuring prior ethical review and approval, and the confidentiality of interviewees and their explicit approval of any quotes.

Though our interview sample was restricted to faculty ISRIA members, we feel this is justified given our aim was to shed light on organisational activities and behaviours (as opposed to theory or principles) of RIA. We recognise that, to a degree, terminology and responses may reflect an already engaged group seeking to contribute to the community of practice in RIA. We appreciate that this engaged group may not be representative of a majority of research organisations, either in terms of capabilities or capacity. Thus, we have made efforts to present our findings in a logical fashion, starting with structural aspects, moving on to procedural aspects and, eventually, the outcomes of conducting RIAs, such that others might be inspired to join this growing community of practice. Wherever possible we have sought to situate responses in context of activities to which other research organisations can relate, and provide specific examples and quotes wherever these do not identify the organisation in question. We would welcome critical feedback and insights from any individuals or organisations who feel motivated to contribute.

## Conclusions

There are very few examples that provide empirical evidence of how research organisations put RIA principles into practice in the scientific literature. From our interviews, we find evidence of the value of RIA, but also a disconnect between published RIA tools and results, and the realities of organisational practices, which tend not to be reported.

Our analysis suggests a number of common areas where research organisations are aligning their practices to optimise research impact and its evaluation. We observed varying structural set ups for conducting RIAs, which included support from senior management and strong leadership, developing a skills base for evaluation, and automating data collection wherever feasible. With respect to processes, we described grassroots efforts to engage researcher communities to articulate and plan for impact, using a diversity of methods, frameworks and indicators for impact assessment, and supporting a learning approach both within and across organisations. Finally, under outcomes of conducting RIA activities, we reported on interviewees’ reflections on the value that RIA has brought to their organisation, to researchers, and to wider communities and stakeholders, including that RIA helps with supporting a dialogue to orient research to impact, underpinning shared learning from analyses of research, and providing evidence of the value of research in different domains and to different audiences.

We suggest three factors that can enable good ‘realistic’ practice in RIA, derived from our analysis, as follows: (1) getting set up for RIA in terms of data, skills, time and supportive leadership able to allocate sufficient resources to developing strategy; (2) working with researchers and other funders and stakeholders collaboratively; and (3) realising RIA benefits such as better data on impact, transparency and the potential to obtain evidence on the value of research.

We conclude that, while theoretical and conceptual RIA models abound, the research organisations’ challenge is to adapt, and experiment with, practical RIA approaches in their own context. Other than the very few notable exceptions that we describe, the ‘science of science’ agenda seems insufficiently embedded in organisational practices if it is to usefully inform RIA. Given research organisations’ key role in shaping research systems, and a growing emphasis on impact, efforts are needed to address this ‘knowledge to practice’ gap.

Assessment of research impact implicitly requires value judgements, on choices of frameworks, indicators, methods, tools, themes and priorities, to name but a few practical considerations. We see from our interviews that research organisations have dedicated time and effort to reflect on how they go about making those decisions and, crucially, engage researcher communities as part of the process. Examples from these organisations that are taking a grassroot, researcher-centric approach to RIA suggest that equal, if not greater, emphasis be placed on strategies to encourage dialogue with researchers and their wider communities around impact, as on evaluative activities to evidence impact. Research organisations benefit from taking a collaborative approach that encourages shared learning as a primary ambition of RIA.

We see a need for investment in skills and supportive structures as well as efforts to make funder datasets more accessible for analysis and publish results to encourage shared learning. We call for research organisations to adapt RIA practices based on clear sight of ‘what works’ in other organisations, as we hope to have begun detailing here. By situating reflections, analysis and further ‘research on research’ into their own working practices, we believe that research organisations can work cooperatively with researchers to orient and optimize research to societal impacts.

## Data Availability

Not applicable.

## Appendix 1

### Search string

List of search terms (and sequence of database searches)

- (translation* adj1 (research or knowledge)) or “knowledge mobili?ation*” or “research into practice” or “translation to health application*” or “translation to patient*” or “translation to health practice*” or “translation to population health impact” or “research impact” or “knowledge into practice” or “populari?ation of research” or “research generated knowledge” or valorization or “value for money” or “social return” or sroi
- metric* or framework* or payback or measure* or “financial return*” or “political impact” or “policy impact*” or “social impact*” or bibliometrics or econometrics or “economic evaluation*” or “cost effectiveness” or “cost benefit analysis” or assessment or evaluation
- (government* or charit* or non-profit* or not-for-profit* or public or health or medic*) adj1 (research or scien*)
- (research or scien*) adj1 (fund* or organi?ation or institution* or grant* or charit* or NGO)
- *1 and 2*
- *3 and 4*
- *5 and 6*
- *remove duplicates from 7*

## Appendix 2

### Interview topic guide

#### Structures and process of research impact assessment (RIA) activities

When did you start undertaking RIA as a formal activity?

Can you briefly describe the methods you currently employ to undertake RIA?

*(frameworks/tools/approaches, based on theory/adapted for own use/developed own?)*

What data do you collect, and how often do you collect it?

What informed your decisions in this regard?

*(literature review/training e.g. ISRIA/own research?)*

Who actually does the work?

#### Outcomes and value of RIA

Can you recall your primary purpose when you first set out to do RIA?

Looking back, what have you found to be the most valuable aspect of doing RIA? *(explore esp. if different from primary purpose/evolved over time?)*

Have you found that undertaking RIA has led to improvements in research translation and impact? If so, do you have any evidence of this?

*(perceived/experiential/substantiated/measured?)*

If RIA helped to identify when research translation wasn’t occurring, what did you do as a result with the information that you gathered?

*(explore organisational links & intentions vs. power to effect change)*

Has RIA facilitated your organisation’s research (+ impact) communications?

*(which audiences, to what effect, any evidence or materials exemplifying RIA?)*

#### Challenges/lessons learned

What were some of the challenges you faced as you implemented RIA practices? *(describe/how overcome/what learned?)*

Is there evidence of others having benefitted from the approach to RIA you’ve taken?

*(organisational or personal reflections/write-ups/reviews/policies?)*

(How) has your approach to RIA developed since you first put it into practice?

*(what informed this?)*

Have you/do you plan to publish materials describing your experiences of RIA?

## Abbreviations

CIHR: Canadian Institutes of Health Research
CTSAs: Clinical and Translational Science Awards
ISRIA: International School on Research Impact Assessment
NIHR: National Institute for Health Research
RIA: research impact assessment

## Footnotes

1 These are freely available under a CC-BY-NC-SA 4.0 license at: https://www.theinternationalschoolonria.com/resources.php
